# Who are the low-risk patients that could benefit from watch-and-wait regarding the neck?

**DOI:** 10.1590/S1516-31802011000500002

**Published:** 2011-09-01

**Authors:** Hugo Fontan Kohler, Luiz Paulo Kowalski

**Affiliations:** I MD. Former Fellow in the Department of Head and Neck Surgery and Otolaryngology, Hospital A. C. Camargo, São Paulo, Brazil.; II MD, PhD. Director, Department of Head and Neck Surgery and Otolaryngology, Hospital A. C. Camargo, São Paulo, Brazil.

**Keywords:** Head and neck neoplasms, Surgical procedures, operative, Mouth neoplasms, Neck dissection, Lymphatic metastasis, Neoplasias de cabeça e pescoço, Procedimentos cirúrgicos operatórios, Neoplasias bucais, Esvaziamento cervical, Metástase linfática

## Abstract

**CONTEXT AND OBJECTIVE:**

The management of clinically negative neck is controversial, with an ongoing debate on the indication criteria and prognostic impact of different types of therapy. The aim here was to compare the results from neck dissection and watch-and-wait, among oral cancer patients who, clinically, did not show any evidence of neck metastasis.

**DESIGN AND SETTING:**

Retrospective analysis in a tertiary cancer center hospital.

**METHODS:**

Patients with epidermoid oral carcinoma were assessed. The inclusion criteria were: primary tumor restricted to the oral/oropharyngeal cavity, no previous treatment, surgical treatment as the first option, clinical/radiological stage N0 and no distant metastasis.

**RESULTS:**

Two hundred and sixty-two patients were analyzed. The length of follow-up ranged from four to 369.6 months and, at the end, 118 patients were alive, 53 had died due to cancer, 84 had died from other causes and 7 had died after the operation. Among the patients who underwent neck dissection, lymphatic vascular embolization (P = 0.009) and tumor thickness (P = 0.002) were significant for regional recurrence, while for the watch-and-wait group, only tumor thickness was significant (P = 0.018). Through recursive partitioning, the patients without adverse prognostic factors and tumor thickness < 2 mm presented compatible results in the two groups.

**CONCLUSION:**

Elective neck dissection seems to be the best treatment option. Patients who are eligible for watch-and-wait constitute a small group that, ideally, is categorized according to the postoperative pathological findings.

## RESUMO

**CONTEXTO E OBJETIVO:**

O manejo do pescoço clinicamente negativo é controverso, havendo um debate corrente sobre os critérios de indicação bem como o impacto prognóstico das diferentes modalidades terapêuticas. O objetivo foi comparar os resultados do esvaziamento cervical com a observação em pacientes com câncer de boca e clinicamente sem evidência de metástases cervicais.

**TIPO DE ESTUDO E LOCAL:**

Análise retrospectiva em hospital terciário, especializado em oncologia.

**MÉTODOS:**

Pacientes com diagnóstico de carcinoma epidermoide de boca foram analisados. Os critérios de inclusão foram: tumor primário restrito à cavidade oral/orofaringe, ausência de tratamento prévio, tratamento cirúrgico como primeira opção, estádio clínico e radiológico N0 e ausência de metástases a distância.

**RESULTADOS:**

Duzentos e sessenta e dois pacientes foram analisados. O tempo de acompanhamento variou de 4 a 369.6 meses e, ao final, havia 118 pacientes vivos, 53 óbitos pela neoplasia, 84 óbitos por outras causas e 7 óbitos pós-operatórios. Nos pacientes submetidos a esvaziamento cervical, a embolização vascular linfática (P = 0,009) e a espessura tumoral (P = 0,002) foram associados significativamente com a recidiva regional, enquanto que nos pacientes somente observados, apenas a espessura tumoral se associou significativamente (P = 0,018). Por meio do particionamento recursivo, aqueles pacientes sem fatores adversos prognósticos e espessura tumoral menor que 2 mm apresentaram resultados compatíveis em ambos os grupos.

**CONCLUSÃO:**

O esvaziamento cervical eletivo parece ser a melhor opção de tratamento. Pacientes candidatos a observação constituem um pequeno grupo e a sua categorização ideal depende de achados patológicos pós-operatórios.

## INTRODUCTION

Management of the neck in patients with oral cancer has been one of the major controversies in head and neck oncology, and most of the discussion has focused on what treatment to administer for patients without clinically evident metastatic disease. For these patients, the incidence of occult neck metastasis may range from 6% to 46%.^1^

The indication for elective treatment of the neck has been considered to be a probability of cervical metastasis of at least 20%,^2^ although reevaluation of this percentage based on decreased surgical mortality and morbidity has been proposed.^3^ These limits are based on conventional pathological evaluation and staining of lymph nodes, but such evaluations have recently been shown to have limitations, in papers using molecular analyses that upstage up to 20% of pathologically N0 patients.^4^

The prognostic impact of therapeutic decisions must also be considered. An elective neck dissection presents risks in the form of postoperative morbidity and mortality and impact on quality of life, but missing a neck metastasis may lead to late recurrences with a significant impact on prognosis.^5^

## OBJECTIVE

To compare elective neck dissection with a watch-and-wait policy, with regard to neck recurrence and survival rates among patients with clinically N0 squamous cell carcinoma of the oral cavity.

## PATIENTS AND METHODS

Patients with primary tumors of the oral tongue, floor of the mouth, inferior gingival rim and retromolar trigone who were treated at Hospital A. C. Camargo, a tertiary cancer center, were enrolled in this study. The data on all patients treated between January 1980 and December 2003 were recovered from the medical records.

The following inclusion criteria were used: histological diagnosis of squamous cell carcinoma, primary tumor restricted to the oral cavity, no previous treatment, treatment with curative intent, surgery as the primary form of treatment, primary tumor staged as T1/T2, clinical/radiological stage N0 and no distant metastasis at diagnosis. The tumors were staged based on the recorded description and pathological report, in accordance with the 2002 AJCC (American Joint Committee on Cancer) classification.^6^

A surgical pathologist dissected all the specimens immediately after removal and three histological slides were prepared from each node.

The statistical analysis was performed using the Stata 11 software for Macintosh (Stata Corp., Texas, United States). Continuous variables were expressed as the mean and standard deviation (SD). Logistic regression was used to assess which factors were significant for the presence of metastatic nodes in the neck. The Kaplan-Meier and Cox regression models were used for recurrence and survival analysis. The classificatory analysis was performed using a recursive partitioning algorithm with the significance level set at 0.05 and a minimum of 20 patients at the knot.

## RESULTS

A total of 262 patients that conformed to the inclusion criteria were analyzed. There were 202 males (77.1%) and 60 females (22.9%), with ages ranging from 23 to 95 years (mean of 58.45 years and SD of 12.0 years). The primary tumor site was the oral tongue in 162 patients (61.83%), floor of the mouth in 73 patients (27.86%), retromolar trigone in 28 patients (10.69%) and lower alveolar rim in 19 patients (7.25%). The clinical T stage was T1 in 99 patients (37.8%) and T2 in 163 patients (62.2%). Neck dissection ipsilateral to the tumor was performed in 166 patients (63.36%); the other 96 patients (36.64%) did not undergo neck surgery. Radical neck dissections was performed on 74 patients (44.58%), modified radical neck dissections on 28 patients (16.87%) and selective neck dissections (levels I to III) on 64 patients (38.55%). A further contralateral neck dissection was performed on 18 of the operated patients (6.86%).

There was a clear time trend relating to the type of neck dissection performed, with increasing proportions of modified radical dissections and selective neck dissections. In 138 patients (83.13%), the neck dissection was removed *en bloc* with the primary tumor and in the remaining 28 patients (16.87%), there was no continuity between the primary tumor resection and the neck dissection specimen.

The decision between observation and neck dissection was significantly correlated with the T stage of the primary tumor and patient gender, but not with age or primary tumor site (Table 1). Blood vessel infiltration was found in six patients (2.42%) and lymphatic embolization in 65 patients (26.21%). Neural infiltration was observed in 73 patients (29.80%). Regarding histological differentiation, the tumors were classified as well differentiated in 178 patients (67.94%), moderately differentiated in 71 patients (27.09%) and poorly differentiated in 13 patients (4.96%). The tumor thickness measured at histological examination ranged from 0.2 to 25 millimeters (mean of 5.81 and SD of 4.33 millimeters). The number of lymph nodes recovered from the neck dissection specimen ranged from 6 to 116 in the homolateral neck (mean of 29.51 nodes and SD of 17.59 nodes).

**Table 1.** Comparison of patients who underwent neck dissection (ND)or observation

Variable	Category	Observation	ND	P-value
Primary site	Oral cavity	90	155	P = 0.905
	Oropharynx	6	11	
Age		60.15	57.48	0.083
Gender	Male	62	140	P < 0.001
	Female	34	26	
T stage	T1	71	28	P < 0.001
	T2	25	138	

The number of retrieved lymph nodes ranged from 8 to 90 (mean of 44.5 nodes and SD of 17.4 nodes) in patients who underwent radical neck dissection and from 6 to 116 (mean of 58.8 nodes and SD of 13.1 nodes) in selective neck dissection patients. Among all the patients who underwent neck dissection, 120 (72.29%) had no metastatic nodes ipsilateral to the primary tumor, while 22 patients (13.25%) presented one involved node, and 24 patients (14.46%), up to eight involved nodes. In the contralateral neck, two patients presented involved nodes. Postoperative radiotherapy was used for 68 patients (25.95%).

The length of follow-up ranged from 4 to 369.6 months (mean of 70.65 and SD of 30.4 months). There were 28 cases (10.69%) of ipsilateral neck recurrence, eight cases (3.05%) of contralateral neck recurrence and three cases (1.14%) of synchronous bilateral recurrence. At the last follow-up, 118 patients were alive and without active disease, 53 patients had died due to disease progression or recurrence, 84 patients had died from other, unrelated causes and seven patients had died following the operation.

Among the patients who underwent synchronous neck dissection, the following factors were significant for the diagnosis of metastatic nodes: size of primary tumor (P = 0.047), histological differentiation (P = 0.002), lymphatic embolization (P < 0.001), neural infiltration (P = 0.045) and tumor thickness (P = 0.018). In multivariate analysis, histological differentiation (odds ratio, OR: 3.78; 95% confidence interval, CI: 1.62-8.78; P = 0.002) and lymphatic embolization (OR: 18.97; 95% CI: 3.98-27.51; P < 0.001) remained significant. Among these patients, there were eight cases of ipsilateral recurrence, eight cases of contralateral recurrence and one case of bilateral recurrence. In univariate analysis, the following factors were significant for neck recurrence: lymphatic embolization (hazard ratio, HR: 1.388; 95% CI: 1.131-2.502; P < 0.001) and tumor thickness (HR: 1.170; 95% CI: 1.027-1.345; P = 0.001). In multivariate analysis, lymphatic embolization (HR: 1.042; 95% CI: 1.034-2.332; P = 0.009) and tumor thickness (HR: 1.069; 95% CI: 1.149-1.316; P = 0.002) remained statistically significant. Among the patients who did not undergo neck dissection, there were 20 cases of ipsilateral neck recurrence, no contralateral recurrences and two bilateral recurrences. All the ipsilateral recurrences occurred at levels I-III. In these patients, the significant factors for neck recurrence were: T stage (P = 0.015), perineural infiltration (P = 0.006) and tumor thickness (P = 0.022). In multivariate analysis, only tumor thickness remained significant (HR = 1.069; 95% CI: 1.012-1.130; P = 0.018). There was a significant difference in mean time that elapsed until neck recurrence between the two groups. Among the patients who underwent neck dissection, the mean time that elapsed until recurrence was 19.75 months and in the observation group, 6.49 months (P = 0.024, Table 2).

**Table 2.** Comparison of neck recurrence time between patients who underwent neck dissection or observation

Treatment	Events	Mean	
Observation	22	6.49	P = 0.024
Neck dissection	17	19.75	

**Figure 1.**
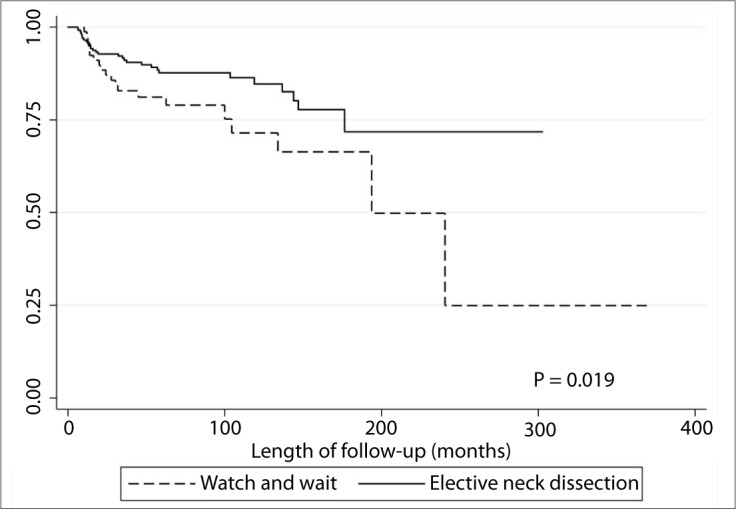
Kaplan-Meier survival curve for patients who underwent either neck dissection or a watch-and-wait policy.

There was a statistically significant increase in the rate of neck recurrence risk among the patients who did not undergo elective neck dissection, in comparison with those who underwent synchronous neck treatment (P = 0.019, Figure 1).

In a multivariate model that included the risk factors for neck recurrence identified in both groups (tumor thickness and lymphatic embolization) and the type of neck treatment and adjuvant radiotherapy, only tumor thickness and synchronous neck dissection were significant (Table 3).

**Table 3.** Multivariate analysis of risk factors for neck recurrence in all groups

	Hazard ratio	95% confidence interval	P
Neck dissection	1		
Watch-and-wait	3.808	1.595-8.391	< 0.001
Tumor thickness	1.126	1.035-1.225	0.006

When we analyzed disease-free survival, the following factors were statistically significant: tumor extent (P < 0.001), T stage (P < 0.001), lymphatic embolization (P < 0.001), neural infiltration (P = 0.039), tumor thickness (P = 0.021) and elective neck dissection (P = 0.023). In a multivariate analysis on survival, lymphatic embolization and elective neck dissection remained significant (Table 4). We also classified the patients through recursive partitioning (RP). This method uses a classification tree and its branches are defined by the variables included in the model. Terminal branches represent RP-derived homogeneous categories according to a specific outcome.

**Table 4.** Multivariate analysis on factors with significant impact on disease-specific survival

Variable		Hazard ratio	95% confidence interval	P
Neck dissection	Yes			
	No	1.587	1.014-2.461	0.032
Lymphatic embolization	No			
	Yes	1.922	1.119-3.303	0.018

**Figure 2.**
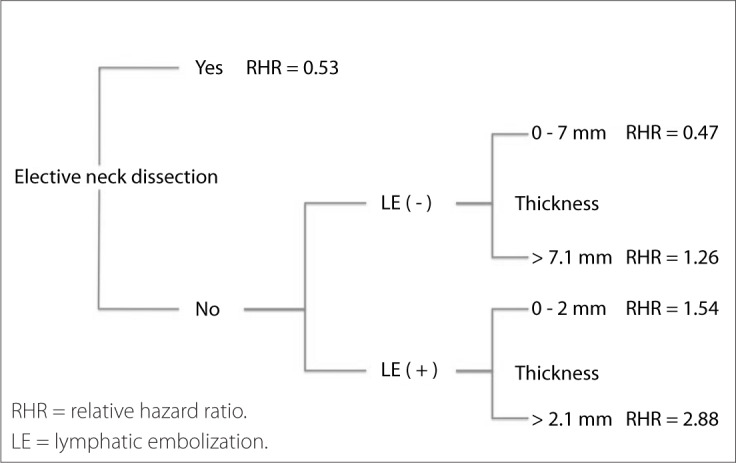
Classification analysis diagram according to survival. Branch splits were performed at a significance level of P < 0.05.

Neck recurrence and disease-specific survival analysis showed that tumor thickness, lymphatic embolization and elective neck dissection were the variables with the best discriminating power for drawing a classification tree (Figure 2).

**Figure 3.**
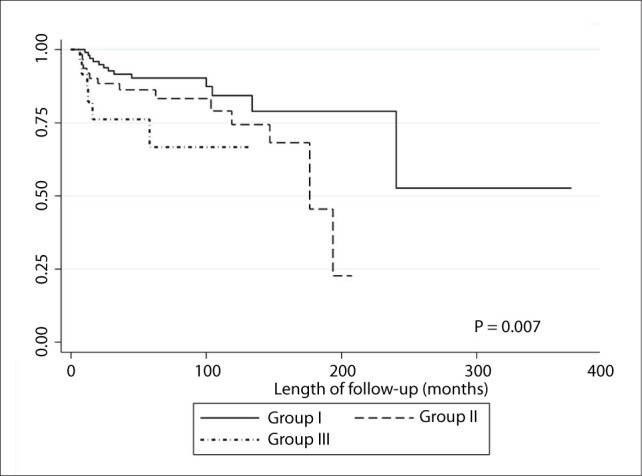
Neck recurrence according to N0 stratification.

**Figure 4.**
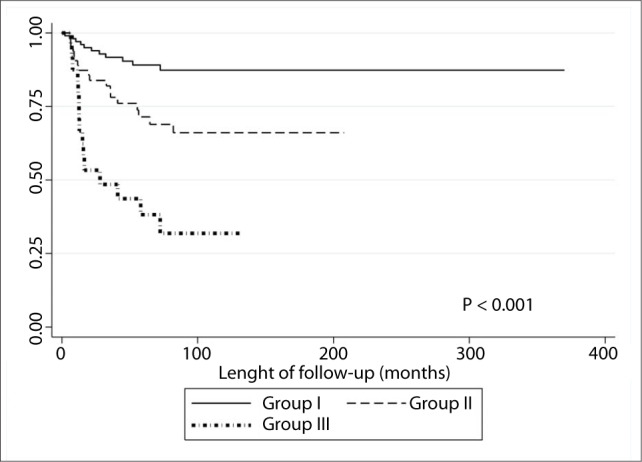
Disease-specific survival of N0 patients according to group stratification.

The first division was elective neck dissection and we decided to group the patients in the observation group into three groups. Group I consisted of individuals with tumor thickness from 0 to 0.7 mm, without lymphatic embolization. This group had a similar relative hazard ratio to that of patients who underwent neck dissection. Group II consisted of patients without lymphatic embolization and with tumor thickness greater than 0.7 mm or with lymphatic embolization and tumor thickness less than or equal to 2 mm. Group III consisted of individuals with tumor thickness greater than 2 mm and lymphatic embolization. There were significant differences between these groups in relation to both neck recurrence rates (Figure 3) and disease-specific survival (Figure 4).

## DISCUSSION

Neck staging is crucial for prognosis definition and treatment planning, since neck metastases are the single most important prognostic factor in head and neck squamous cell carcinoma.^7^ Occult neck metastases have a significant impact on survival.

In a study on patients with clinically node-negative necks, the rate of occult metastases was 50% and these patients had significantly worse survival (P < 0.001).^8^ Also, the diagnosis of node metastases and the presence of extracapsular spread are considered to be an indication for adjuvant treatment.^9^ On the other hand, a neck dissection may avoid unnecessary adjuvant treatment and spare the use of radiotherapy.^10^

Surgery alone may achieve a control rate on pN0 necks of 75% and may compare favorably with radiation therapy.^11^ Neck metastases have been linked to certain factors. Tumor thickness has been significantly linked to postoperative upstaging of the neck, and a positive correlation between tumor depth and T staging has also been demonstrated.

In one study, a cutoff point of 4 mm was suggested for risk stratification, although those authors suggested that for oropharyngeal tumors, a lower cutoff point might be required.^12^ This finding had been previously demonstrated in another study that suggested that this cutoff point could be used in making the decision regarding elective treatment of the neck for patients with oral tongue carcinomas.^5^ In another report, a cutoff point of 3 mm, for moderate or poor differentiation, cases of perineural invasion and lymphovascular permeation had a significantly higher incidence of occult neck metastases.^13^

Simultaneous use of tumor thickness and histological differentiation has also been proposed for stage I and II tongue carcinomas. Kurokawa et al. suggested that tumor depth > 4 mm and moderately differentiated carcinoma should be definitive indications for neck dissection.^5^

Management of N0 necks may fall into three categories: elective neck dissection, radiotherapy or observation. The choice between radiotherapy or neck dissection will depend essentially on the treatment for the primary tumor. An approach based on location and stage of the primary tumor was shown to be effective, with 9% development of neck recurrences in early-stage oral cancers.^14^

Using a decision-analysis approach, Song et al. demonstrated that neck dissection was the preferred management for early-stage tongue cancer in clinical N0 necks. These authors stated that the incidence of neck recurrences was high and that pathological analysis was more precise than imaging methods and allowed for improved definition of postoperative chemoradiotherapy. However, if the risk of neck metastasis was lower than 0.17 and the salvage rate higher than 0.73, watchful waiting would be an appropriate choice.^15^

The use of irradiation, although with similar control rates when compared with neck dissection, was found to have significantly higher incidence of adverse side effects.^16^

In patients with early-stage oral carcinoma, elective neck dissection was seen to be a significant factor for recurrence (8% versus 26.8%; P = 0.001) and survival rates (P < 0.01), thus suggesting that elective neck dissection was superior to observation alone. A significant benefit regarding survival and neck recurrence rate was also observed in another series of 380 patients with early-stage oral tongue squamous cell carcinoma.^17^

The importance of surgical staging for treatment planning should also not be underestimated, with 40% stage migration in a series of patients with T1-T2 N0-N1 oropharyngeal cancers.^18^

This evidence goes against a recent report that showed that there was no survival advantage for patients who underwent neck dissection, in comparison with a watchful waiting policy.^19^

In a prospective, randomized clinical trial comparing elective neck dissection and observation in cases of early stage oral tongue carcinoma, the five-year disease-specific survival was comparable, with no statistically significant difference between the two groups. The neck recurrence rate was higher in the observation group but because of the strict follow-up schedule, salvage was possible in all cases. That trial supported the use of watch-and-wait and a strict observation schedule.^20^

This treatment choice was also supported by another report that outlined a sensitivity analysis on neck metastasis in cN0 patients.^21^

## CONCLUSION

Our data show that clinical N0 patients with oral cancer are a heterogeneous population with different rates of neck recurrence and disease-specific survival. Our decision tree approach was able to stratify them into three distinctive groups and show the importance of neck dissection. For the patients who did not undergo neck dissection, only a defined set of individuals had comparable regional recurrence rate and survival.

This stratification could only be performed using pathological variables that became available after the definitive pathological report had been produced, thus limiting its applicability. Therefore, elective neck dissection seems to be the best treatment option. Patients eligible for watch-and-wait constitute a small group, which is ideally assessed according to the postoperative pathological findings.
